# The views of the elderly on the impact that HIV and AIDS has on their lives in the Thulamela Municipality, Vhembe District, Limpopo province

**DOI:** 10.4102/curationis.v38i1.1166

**Published:** 2015-06-09

**Authors:** Vhudivhusi J. Singo, Rachel T. Lebese, Thelma X. Maluleke, Livhuwani H. Nemathaga

**Affiliations:** 1 Department of Public Health, University of Venda, South Africa; 2 Department of Advanced Nursing Science University of Venda, South Africa; 3 Human Science Research Council, South Africa

## Abstract

**Background:**

HIV and AIDS have a devastating impact on the lives of elderly people, particularly so because they are often expected to take care of their terminally ill children and assume the responsibility of looking after children orphaned by AIDS - in most cases with very little resources.

**Objectives:**

The study sought to achieve to describe the views of elderly people regarding the impact of HIV and AIDS on their lives, to determine the challenges that elderly people living with HIV or AIDS (EPLWHA) face in their daily lives, and to gain a sense of the coping strategies they use to overcome the obstacles they face in relation to HIV and AIDS. Ethical issues, such as permission to conduct the study, informed consent, confidentiality and anonymity, withdrawal of participation and measure to ensure trustworthiness, were ensured.

**Design:**

This was a qualitative, explorative, descriptive study. Participants were interviewed using an interview guide. Information provided by the participants was captured on a tape recorder and analysed using open coding, and thereafter collated into themes, categories and sub-themes.

**Results:**

The study findings revealed that HIV and AIDS have serious negative impacts on the lives of elderly people, particularly those living in poverty. The following key areas in relation to EPLWHA were established: psychological or emotional health, as well as household and socio-economic burdens. Considering the role that elderly people play in the community in so far as HIV and AIDS are concerned, primary health promotion and social welfare programmes should be directed at educating all elderly people and their service providers on how to cope with the health and social problems related to HIV and AIDS.

## Introduction

Infections with the human immunodeficiency virus (HIV) and acquired immune deficiency syndrome (AIDS) have a devastating impact on the lives of elderly people, but remain underreported (Avert 2008). It is further believed that there is an urgent need to conduct research on the effects of HIV and AIDS on elderly people in many African countries because of their vulnerability resulting from lifetime hardships, susceptibility to malnutrition, poverty and chronic diseases. Thus, HIV and AIDS are now posing an additional burden to the aged (Centre on Aging Studies 2007).

Elderly people require support and expect to be looked after by their children and significant others. However, due to the HIV pandemic, the opposite usually happens (Avert 2008). Elderly people are often required to support and care for their sick and dying children and grandchildren. This poses a greater risk to them as they play the role of caregivers without much knowledge of the disease with which a family member is infected. As a result, they are not familiar with the ways of protecting themselves from the disease, and in most instances, without even the basic resources to manage the sick person (Avert *ibid*). In this era of HIV, many elderly people are either affected by AIDS or infected with HIV. Despite their poverty, they care for their dying children and/or orphaned grandchildren (Avert *ibid*).

Informal caregivers of people living with HIV or AIDS (PLWHA) are mainly elderly family members. These caregivers usually display depressive symptoms, anger and anxiety. The symptoms are more pronounced amongst low-income families and these have detrimental effects on the physical health of the elderly caring for their sick family members (Noumbissi 2003). Poor families have insufficient resources to care for PLWHA and hence there is a high risk of elderly people infecting themselves when caring for a sick family member. On the other hand, the increasing numbers of children orphaned by AIDS affect the living conditions and well-being of the elderly who have to care for their sick children and grandchildren (Noumbissi *ibid*).

## Problem statement

HIV places enormous stress on infected individuals and their families. In particular, family members are confronted with the demands of caring for the seriously ill, as well as the trauma of death. The impact of HIV is worsened by the stigma associated with the condition, the material and psychological strain of coping with HIV-related deaths and bringing up orphans (Avert 2008). Elderly people living with HIV or AIDS (EPLWHA) in the Vhembe District experience enormous stress. In many cases, elderly people are faced with caring for the sick and looking after orphaned grandchildren. There are currently several programmes that address HIV infection in young people. However, very little is done for the elderly who are infected with HIV or affected by AIDS, and very few services are available to assist and train them on how to cope with the disease burden. Therefore, it was considered important to conduct this study to explore and describe the impact of HIV and AIDS on EPLWHA.

## Purpose of the study

The purpose of this study was to explore and describe the views of the elderly in Thulamela Municipality of Limpopo Province on the impact of HIV and AIDS on their lives.

## Research objectives

The objectives of the study were to:

- Identify challenges faced by the elderly people infected with HIV and affected by AIDS.
- Describe coping mechanisms used by the elderly when dealing with HIV and AIDS.
- Describe the elderly people's experiences related to finding out about the HIV status of family members or themselves.
- Explore the elderly people's experiences of caring for an HIV or AIDS patient.

## Definition of concepts

### AIDS

AIDS is a syndrome of opportunistic diseases, infections and certain cancers, each or all of which has the ability to kill the HIV-infected person in the final stages of the disease (Van Dyk 2008). AIDS is also called an incurable decease caused by a germ (Paine 1990).

### HIV

HIV is a blood-borne virus. It is transmitted primarily through sexual contact, needle sharing, transfusion of tainted blood products, and perinatal transmission (i.e. through the birth canal, or breastfeeding). It is a virus that knows no boundaries and can be found in persons of all ages, races socio-economic classes and sexual orientations (Centre on Aging Studies 2007).

### Elderly people

Elderly people are normally individuals over the age of 60, characterised by vulnerability such as poor physical health, mental illness, poverty, inadequate education, lack of social support and dysfunctional family (Huber *et al.* 2008). In this study an elderly is defined as a person above 50 years affected by AIDS or infected with HIV.

### Impact

Impact is described as significant consequences, for example, family and friends are put on an alert and may respond either by renewed efforts to help the individual or by further emotional isolation of the affected individual because of their own heightened anxiety (Kastenbaum 1998). Impact in this study will refer to the consequences, which could be challenges, benefits, gains or coping strategies that the people may experience as a result of HIV and AIDS.

## Significance of the study

It is anticipated that the study would contribute to the identification of challenges faced by the elderly affected by AIDS or infected with HIV. The recommendation may assist health workers in planning how to assist elderly people in this situation.

## Research design

Research design is a logical strategy for gathering evidence about the knowledge desired. It must be efficient, which means it must actually yield the knowledge desired (De Vos *et al*. 2005). The study design was qualitative, exploratory and descriptive in nature. Qualitative research is an approach that does not usually provide the researcher with a step-by-step plan or a fixed recipe to follow (De Vos *et al. ibid*). Qualitative methods collect information in the form of words that give an in-depth understanding of the nature of what people experience. In this study, the researchers were interested in the in-depth descriptions of the range of emotions, thoughts and coping strategies that people exhibit in particular situations. Qualitative methods allow participants to speak about their experiences in their own words (De Vos *et al*. *ibid*). The focus of this qualitative study was to gain insight into the personal experiences of the participants (elderly people) regarding the impact of HIV and AIDS on their lives. The data were collected in the natural settings of the participants, which were their home environments. The study also sought to understand the deepest experience of EPLWHA. Social workers’ views on the impact of HIV and AIDS on the elderly were explored and described. Data from social workers were collected in their respective offices. Explorative studies were conducted to explore the topic in question or to provide a basic familiarity with that topic. This approach is typical when a researcher examines a new interest or when the subject of the study is relatively new. Such studies are also done to satisfy the researcher's curiosity and desire for a better understanding and to determine the priorities of future research (Babbie & Mounton 2001). The use of open-ended questions and the probing of participants’ answers allowed for flexibility to further explore the study topic. The researcher was able to move beyond the limits of the present questions based on expected findings from the literature to explore the impact of HIV and AIDS on the elderly.

### Population and sampling

The study population comprised all elderly clients who were 50 years old and older, both males and females, receiving grants because they are either infected with HIV and/or affected by AIDS. These were clients in the records of social workers within the Social Development Section of the Thulamela Municipality.

The sampling was purposive because the researcher selected elderly clients who are documented on social workers’ record books. Those were people 50 years and older, infected with HIV and/or affected by AIDS, and residing in the villages and townships in the Thulamela Municipality. Participants were selected based on the following criteria:

- Receiving foster care grant for children orphaned by AIDS.
- Taking care of an adult who is receiving a grant because of HIV or AIDS.
- Receiving a grant because of HIV or AIDS.
- Participants were selected from three social work sub-offices, viz. Thohoyandou, Makwarela and Tshilidzini. Clients who fulfilled the requisite criteria were identified − a total number of 12 were reached and the sample was determined by data saturation.

### Data collection

Data collection is a process in the research project that involves engaging with a target sample or population from which data are collected (David & Sutton 2004). Data collection is also defined as a series of interrelated activities aimed at gathering good information to answer emerging research questions (Creswell 2007). The researcher used an interview guide to collect data.

### Data analysis

The researcher analysed the data in a qualitative manner using Tesch's method (cited in Creswell 2003). Data was broken into discreet parts, and then closely examined and compared for similarities and differences. The questions asked were reflected in the data and themes, and categories and subcategories were developed from data.

## Ethical consideration

Ethical standards were based on the Democratic Nurses Association of South Africa's (DENOSA) ethical standards for nurse researchers (DENOSA 1998).

Permission to sample the elderly people was obtained from the district office and Limpopo Province Provincial Health Department. Each elderly person was provided with sufficient and understandable information regarding participation in the study before signing the consent form. Confidentiality and anonymity were ensured by protecting the participants’ identity, privacy, self-worth and dignity by not indicating the subjects’ names on the questionnaires. Participants were given every assurance that they were free to discontinue their participation at any time without being required to offer an explanation. The participants were ensured that their participation or non-participation will not have any impact on the service they received, meaning the participants were told that they could refuse to participate in the study without any penalty.

### Measures to ensure trustworthiness, reflexivity, transferability and conformability

Trustworthiness, another approach to clarify the notion of objectivity as it is manifested in qualitative research, is found in the highly influential work of Lincoln and Guba (1985). The researcher achieved credibility by making sure that the participants understood the questions. This was done by reframing the questions whenever the respondents showed misunderstanding. Reflexivity was ensured by interviewing participants in different venues and settings. Participants were given sufficient time to respond to questions and this ensured prolonged engagement. Transferability was ensured by requesting assistance from colleagues to purposefully select the participants with the needed characteristics. This was done in order to ensure that the results suited other participants with the same characteristics. The characteristics and methods were also clearly described. Conformability was achieved by prolonged engagement with participants during in-depth individual interviews.

### Participants’ characteristics

The findings were gathered from 12 elderly people aged from 50 years to 83 years, in social workers’ records infected with HIV and/or affected by AIDS. The participants were categorised as follows:

- Taking care of infected adults.
- Taking care of children orphaned by AIDS.
- EPLWHA.

## Presentation and discussion of the findings

Table 1 summarises the themes, categories and subcategories that emerged from the analysis of raw data of the general participants. General participants expressed different experiences related to the impact of HIV and AIDS. Five major themes emerged from the data with appropriate quotes from raw data and relevant literature was used to substantiate the themes. The following themes emerged:

- The experience of general participants related to finding out about their own or family members’ HIV status.
- The general participant's experience of caring for an HIV or AIDS patient.
- Challenges experienced when looking after PLWHA or children orphaned by AIDS.
- The impact of HIV and AIDS on the family.
- Coping strategies used by the elderly.

**TABLE 1:** Themes, categories and subcategories of the impact of HIV and AIDS on EPLWHA, according to the elderly infected by HIV or affected by AIDS.

Theme	Category	Subcategory
1. Experiences related to finding out the HIV status of a family member	1.1 Difficulties in relation to finding out about own or family member's HIV status	1.1.1 Finding out after the death of their children or grandchildren
		1.1.2 Verbal and physical abuse of the elderly by HIV-positive patients
	1.2 Positive ways of finding out about the HIV status	1.2.1 Status revealed to the elderly by the healthcare worker
		1.2.2 Finding out about own HIV status after voluntary testing
2. The general participants’ experiences of caring for an HIV patient	2.1 Difficulties related to caring for HIV-positive patients	2.1.1 The experience is tough and stressful
		2.1.2 Poverty worsens the situation
	2.2 Positive experiences related to caring for an infected family member	2.2.1 Support from infected to affected family member
		2.2.2 Understanding the treatment of HIV-positive patients
3. Challenges experienced when looking after		
PLWHA or children orphaned by AIDS	3.1 Financial challenges	3.1.1 Lack of income
		3.1.2 Cut off from the disability grant
		3.1.3 Fear that funeral policies might not be enough
	3.2 Health challenges	3.2.1 Due to stress, chronic conditions are worsened
		3.2.2 Skipping of meals due to stress
	3.3 Social challenges	3.3.1 Verbal abuse
		3.3.2 Estate disputes
		3.3.3 Uncontrollable children and their elder siblings
4. Impact of HIV and AIDS on the family	4.1 Disorganisation of the family	4.1.1 Elderly people moving to the houses of their late children or grandchildren moving in with their grandparents
	4.2 Sibling disputes	-
5. Coping strategies used by the elderly	5.1 Positive coping strategies	5.1.1 Department of Health and Social Development
		5.1.2 Churches
	5.2 Negative coping strategies	5.2.1 Neither friend nor relative support

## Theme 1: The general participant's experiences related to finding out about own or family member's HIV status

All participants expressed their experiences in finding out their own or a family member's HIV status. Three categories were identified from this theme, namely: difficulties in relation to finding out about own or family member's HIV status, positive ways of finding out about the HIV status and verbal abuse.

### Difficulties in relation to finding out about own or family member's HIV Status

The majority of the participants expressed their views on their difficulties in finding out about the status of family member or themselves.

The results indicated that elderly people usually only find out about the status after the patient's death or when they are terminally ill. An HIV-positive 63-year-old said:

‘I never knew about her status, but I think I now know. The other time I started to develop some sores, so I decided to be tested and I tested positive […] I believe she infected me because I took care of her without protecting myself, because I did not know [*about*] her problem. My husband died 31 years ago − he cannot be the one; she is the one.’

One participant expressed her feelings as follows:

‘I started to hear rumours that my son, who stays in the same neighbourhood, is very sick. When I asked him, he told me he has [*sic*] [*the*] flu. He died and after few months his ex-wife died, then followed the new wife. With my son I used [*sic*] to believe that he slept with a woman who was on [*her*] menstrual period. One day community-based workers came looking for one of his children. I asked more question[*s*] [*and*] the child himself confessed that he got infected because he was taking care of his mother. That's how I found out that they all died of AIDS.’ (An affected 69-years-old)

The findings are supported by Avert (2008) wherein elderly people are required to support and care for their sick and dying children and grandchildren, exposing them to risks of infections. In the study by Shiluvane *et al.* (2011), tuberculosis (TB) was perceived as confirmation that the individual has HIV. This contributed to stigmatisation of the disease and the people caring for patients infected with HIV, which could be a motivating factor for non-disclosure.

### Verbal and physical abuse of the elderly by HIV-positive patients

The results showed that even if the elderly suspect that a family member is infected, they hesitate to confront them because of fear of abuse, either verbally or physically. An angry 70-year-old said:

‘The day that my daughter found me admitted with her son, she caused a scene, screaming at me, indicating that I told the doctors that she has multiple sexual partners. As such I have told the medical staff about her HIV-positive status. Remember, I was not even aware about her HIV status, but she was accusing me of gossiping about her HIV status. I do not know what was wrong with my daughter − she used to assault me verbally.’

According to Avert (2008), elderly people indicated that they have experienced either verbal or physical abuse from HIV-infected persons, as well as from their terminal ill children.

### Positive way of finding out about the HIV status

The study has revealed that some adults understand that they should tell their parents about their HIV status, which is how the elderly would know. A 69-year-old participant, showing signs of appreciation, said:

‘The other day […] I got a message that one lady, who is my neighbour and health worker, is calling me to [*sic*] her place and when I arrived she disclosed the status of my daughter. I took it well and knew that she needs my support.’

According to the literature, disclosure of someone's HIV status to their partners is associated with breakdown in social support, complicated by the unpredictable behavioural patterns, which poses a considerable dilemma for public health practitioners. Whether emphasis is on confidentiality or encouraging HIV disclosure, it has been suggested that emphasising confidentiality may safeguard social support structures, but this is at the risk of discordant partners being infected under a professional's watch (Chew, Beng & Mun 2012).

A 59-year-old woman, looking very sad but she did not disclose her status, said:

‘I had flu for two weeks with a headache and diarrhoea and these [*sic*] are […] all symptoms that I have heard [*about*] on the radio, so I requested […] a test.’

These findings are in contradiction with literature, which indicates that the majority of elderly people lack knowledge about agencies and available services (Avert 2015). There is therefore a need for awareness programmes that will target doctors and healthcare professionals to try to move away from only targeting young people in HIV and AIDS prevention and education programmes as this may be denying many elderly people health education and early diagnosis (UNAIDS 2005).

## Theme 2: The general participant's experiences of caring for an HIV or AIDS patient

### Difficulties related to caring for HIV-positive patients

#### The experience is tough and stressful

According to the results, EPLWHA are likely to witness their children and/or grandchildren being terminally ill and even die in their presence. Findings revealed that the majority of them took care of more than one patient, and some have already experienced more than one death of their loved ones. A 60-year-old, looking very tired, expressed her feelings as: ‘It is tough because you don’t even know what to do with a patient or what to give.’

A 72-year-old participant, looking sad, had this to say:

‘Keeping time of when to take the [*sic*] medicine is tough. Remember, it is not temporary − it is for the rest of her life. She needs to wake up very early so that she eats [*sic*] breakfast, take [*her*] medication and then go to school.’

According to Avert (2008), older caregivers are under serious financial, physical and emotional stress due to their caregiving responsibilities, which supports the findings of this research. Thomas (2002) also indicated how caregivers experience physical strain and emotional consequences when caring for patients and this leads to inability to provide adequately for the patient. The caregivers also become fatigued by caring for the patient for a long time and some eat less or not at all when the patient is seriously ill (Thomas *ibid*).

#### Poverty worsens the situation

The study reveals that poverty is making the already difficult situation even more demanding. The only male, who was 65 years old, looking very worried, said:

‘I receive the old age grant [*while*] being sick at the same time; my wife is [also] not working due to health issues. As a result, my grant is expected to cover […] the needs [*of*] the whole family − remember my wife and I need food before we take medication. I do not believe that the grant money is [*meant*] to maintain the whole family, but that's what is happening in my own house. On the other hand, I have to make sure that our funeral polices are in place, because we are the living dead.’

According to Avert (2008), most elderly people are particularly vulnerable to poverty when they retire. When HIV-infected children die, elderly people must find money for burials, as well as the strength to care for those left behind. Should the patient be the breadwinner and eventually lose their job, the family may find it hard to make ends meet (Bezuidenhout 2008). A similar trend has been found in the study by Sukumani *et al.* (2011), where caregivers are sometimes expected to buy food, toiletries and other necessities for the patients, which is the problem for them as most of them depend on child pension grants.

### Positive experiences related to caring for an infected family member

The study's findings revealed that there are some positive experiences in relation to taking care of an infected family member, such as support from infected to affected family members (who have personally gone through the experience and therefore understand the treatment required for HIV-positive patients).

#### Support from infected to affected family member

The study revealed that for those EPLWHA who also have another family member infected, good support is given because they understand each other better because of their mutual experience. A 65-year-old male (the only male participant in the study) said:

‘Immediately when my wife and I found about our status, we went […] for treatment. Now we are each other's treatment supporter[*s*] − we keep reminding each other about when [*sic*] to take [*the*] treatment. We also encourage each other not to skip [*sic*] the treatment.’

This is how a 68-year-old woman, not showing any signs of anger about her status, puts it:

‘I started to get sick too often and my daughter advised me to get [*sic*] tested − she even accompanied me to the clinic. I tested positive, [*but*] she supported me. We always encourage each other to take [*sic*] the treatment because if we don’t there are [*sic*] going to be negative results.’

Ateka (2006) found that mothers disclose more often to their daughters than to their sons. Chimwaza and Watkins (2004) also described how participants displayed love for their patients and felt that it was their duty to love them and care for them, even if they did not wish to do that.

#### Understanding the treatment of HIV-positive patients

Participants described how having an understanding on the treatment seemed to reduce their stress. A 67-year-old participant said:

‘It is easy if one follows the treatment. In fact, [*sic*] they let you choose your own time: if you choose 7, it must be at [*sic*] 7 am and 7 pm. Sometimes we fail to meet [*sic*] the time by [*a*] few minutes, but we know that it should not [*sic*] be a tendency as it can be dangerous to us.’

According to Hejoaka (2009), the necessity of routinely taking medicines is one of the most common constraints faced by patients and treatment supporters. Since the effectiveness of antiretroviral drugs is dependent on the regularity of the treatment, precise hours are specified to ensure a patient's adherence.

## Theme 3: Challenges experienced when looking after PLWHA or children orphaned by AIDS

The participants explained the challenges that they were facing whilst taking care of people living with HIV/AIDS or those orphaned by the disease and these were identified as financial-, health- and social-related challenges. One participant, 58 years old and desperately waiting for her pension grant, explained her situation:

‘I do not have an income − not even a grant for me to eat. It means I must first do [*a*] piece job. Mostly, I depend on my unemployed daughter who is married. I do not even know how long she will be able to assist as she depends [*sic*] on her husband.’

Knodel and Wassana (2004) found that EPLWHA are deprived of the support that the adult child, who is terminally ill or died, would have provided, and they end up with no other source of income other than social grants. Rowe *et al.* (2005) also found in their studies that family members were expected to provide special food for patients, such as eggs, meat, milk, soft drinks, oranges and vegetables, which the caregivers could not afford.

Most participants expressed their fear of being cut off from social grants, leading to insufficient funeral policies.

### Health challenge

A 69-year-old expressed her feelings as follows: ‘Ever since I started feeling [*the*] pressure of [*sic*] taking care of my daughter, [*I*] am afraid I may even die due to high blood pressure.’ Another participant, 73 years old and looking very sad, explained: ‘I can no longer do heavy duties as I used [*sic*] to do. I have asthma and sugar diabetes − now that [*I*] am HIV-positive the situation is worse.’

Chanda and Gosnell (2006) indicated that family members sometimes lose interest in continued supervision and care for patients. Munthali's findings (cited in Chanda & Gosnell *ibid*) correspond with those of Sagbakken, Frich and Bjune (2008) that the chronic nature of the disease and prolonged treatment protocol can affect compliance to treatment, even with family support. Illnesses such as heart diseases, depression, high blood pressure, arthritis and Alzheimer's are more prevalent in older persons, who are therefore more likely to be taking medicines for long-term illness or as a preventative measure for a pre-existing condition (Avert 2008).

Most elderly people focus on caring for their children and grandchildren, neglecting themselves,, mostly going without food in order to provide for others, and risk becoming ill. This may result in malnutrition and rapidly failing health (Today’ s Research on Aging 2007).

### Social challenges

Social challenges faced by EPLWHA involved verbal abuse, estate disputes and being left with uncontrollable children and their older siblings.

A 70-year-old participant, looking very sad, said:

‘My daughter always uses painful words towards [*sic*] me − each time I fail to do something for her, she tells me it is because I want her dead. What I know is that she does not like me − she thinks I am bad. She believes people from [*the*] outside love her [*the*] most. She wants to go away from me, while I want her to be close because I know that if she goes, she will not be able to take care of herself.’

Avert (2008) findings indicated that EPLWHA suffer verbal and physical abuse from HIV-infected family members, including from their terminally ill children.

This is how a 65-year-old participant expressed her feelings:

‘I do not know what to do with the older [*sic*] grandchild. She is no longer staying at home. She moves from one village to the next. If I follow her, she runs away to the next village. I even hear that she has several boyfriends; she is using substances and stealing. It is just that I cannot shift this responsibility – [*there is*] nowhere else I can take them.’

Bezuidenhout (2008) found that family disorganisation due to the loss of parents may prompt members to engage in deviant acts, such as truancy, prostitution, drug abuse, suicide attempts and wife and children battering. Family members may experience fear, anxiety and feelings of guilt about their situation.

## Theme 4: Impact of HIV and AIDS on the family

All of the participants discussed the impact that HIV and AIDS have on the family − issues such as family disorganisation and sibling disputes. Elderly people are forced to move to the houses of their late children or grandchildren have to move in with their grandparents.

This is how a 71-year-old participant puts it:

‘When my daughter got terminally ill, I had to move to her place so that I was [*sic*] able to take care of her until she died. Now [*I*] am back again for her children. I was supposed to be staying in my own place, but [*sic*] I can’t − who will stay with these [*sic*] poor orphans?’

Bezuidenhout (2008) supports this finding by indicating that HIV, AIDS and TB endanger the life of an individual and could result in the death of a family member, like the breadwinner. The loss of both parents results in family disorganisation. Often children infected with HIV return to their parents’ home during the final stages of the illness (Help Age International 2004).

A 56-year-old participant, who appeared very sad, stated:

‘Now I am neglecting other children's financial needs in order to cover all her expenses. I am taking other children‘s school fees money to assist in [*sic*] her needs, because her needs are too expensive, such as medicine and food. My other children always complain that it seems as if she is my only child, and that's causing tension in my house. I even suspended other projects just to focus on her, because I can’t afford all my family's needs.’

These findings are supported by Barnett *et al.* (2000), who found that in families affected with HIV and with little resources, social relationships are often strained as they are expected to share their little available resources.

## Theme 5: Coping strategies used by the elderly

All participants expressed their ways of coping with the challenges caused by HIV and AIDS – these include both positive and negative strategies. Positively, the elderly get support from the Department of Health and Social Development and the church, as reflected by participants. One 68-year-old HIV-infected participant said: ‘I have no other support, except that from social workers, clinic and hospital. Social workers give us food parcels, when I get sick I go to the clinic or hospital.’

The pivotal roles that the National Departments of Social Development and Health have concerning HIV and AIDS were corroborated. The National Strategic Plan on HIV and AIDS, and sexually transmitted infections (STI) affirmed the responsibility that these lead departments should jointly take in reducing the impact of HIV and AIDS on communities (National Department of Health 2007).

This is how a 65-year-old participant expressed her feelings:

‘My grandchildren have a tendency of fighting − each time they start, I begin to sing church songs and it's helping me a lot. Since [*I*] am not able to guide the children, the pastor sometimes visits us and tries to guide them on sexual-related issues and drinking alcohol. I really see it helping.’ (Participant 4)

The role that the church is playing in offering support is confirmed by other literature. Normally, church members visit families infected with HIV and/or affected by AIDS and pray for them. However, the view is that churches are still to come to terms with the complex challenges of HIV and AIDS. For example, churches do not have activities that openly address HIV and AIDS. It is clear that churches took a punitive approach, regarding infection with HIV as the result of bad sexual behaviour (Campbell *et al.* 2007).

The study's findings also revealed that none of the participants are receiving support from a close relative. This is how a participant, looking very worried, expressed her experiences:

‘My older [*sic*] sister's daughter assisted me with the burial of my late daughter, but she told me to remember that she is not my relative. She told me she is only my sister's child. As a result I feel that am alone.’ (Participant 7, 59 year old)

This finding is supported by Pallangyo and Mayers (2009), who also found that sometimes relatives avoid assisting as they are afraid that they may end up taking up the entire responsibility.

## Recommendations

The following recommendations were conceptualised to ensure that proper and sufficient services are rendered to EPLWHA and PLWHA:

- The role that elderly people play in this era of the HIV pandemic should be recognised. They deserve to be acknowledged by the government and other sectors of society.
- Elderly people involved in HIV and AIDS care should be empowered with contemporary parenting skills as there is a serious generational gap between them and their grandchildren, and this contributes to widespread misunderstanding and conflict in grandparent-headed households.
- Elderly people who qualify for the disability grant should be given the explanation and terminology they can understand, that is how are they going to receive the grant, and why and when it will be cut off.
- The elderly should be incorporated into job settings that cater for their age groups so that they can supplement their government grants and lessen financial burdens associated with caring for HIV-infected persons.

### Limitations

As the study involved a condition that is stigmatised, some of the selected participants hesitated to be recruited. The problem of disclosure was evident as the researcher was regarded a stranger by some of the participants. The researchers also did not manage to obtain participants from the Malamulele area, which is also part of the Thulamela Municipality.

## Ethical clearance number

The Institutional Health, Safety and Research Ethics granted permission on 22 October 2010. Ethical clearance number SHS/10/PH/004.

## Conclusion

There is a need to involve spiritual and traditional leaders as they have a huge influence on elderly people and receive a lot of respect from them. Elderly people who are not infected or affected need to be encouraged to form peer support groups. Family members should also be involved to support the infected and affected to reduce stigmatisation.
